# A Single-Use, In Vitro Biosensor for the Detection of T-Tau Protein, A Biomarker of Neuro-Degenerative Disorders, in PBS and Human Serum Using Differential Pulse Voltammetry (DPV)

**DOI:** 10.3390/bios7010010

**Published:** 2017-02-19

**Authors:** Yifan Dai, Alireza Molazemhosseini, Chung Chiun Liu

**Affiliations:** 1 Department of Chemical & Biomolecular Engineering and Electronics Design Center, Case Western Reserve University, 10900 Euclid Avenue, Cleveland, OH 44106, USA; yxd176@case.edu; 2 Dip. Chimica Materiali e Ing. Chimica “Giulio Natta”, Politecnico di Milano, Via Mancinelli 7, 20131, Italy; axm1058@case.edu

**Keywords:** T-Tau protein detection, differential pulse voltammetry, [Fe(CN)_6_]^3−/4−^ redox probe, 3-mercaptopropionic acid (MPA)

## Abstract

A single-use, in vitro biosensor for the detection of T-Tau protein in phosphate-buffer saline (PBS) and undiluted human serum was designed, manufactured, and tested. Differential pulse voltammetry (DPV) served as the transduction mechanism. This biosensor consisted of three electrodes: working, counter, and reference electrodes fabricated on a PET sheet. Both working and counter electrodes were thin gold film, 10 nm in thickness. Laser ablation technique was used to define the size and structure of the biosensor. The biosensor was produced using cost-effective roll-to-roll process. Self-assembled monolayers (SAM) of 3-mercaptopropionic acid (MPA) were employed to covalently immobilize the anti-T-Tau (T-Tau antibody) on the gold working electrode. A carbodiimide conjugation approach using N-(3-dimethylaminopropyl)-N’-ethylcarbodiimide hydrochloride (EDC) and N–hydroxysuccinimide (NHS) cross-linked anti-T-Tau to the carboxylic groups on one end of the MPA. A T-Tau protein ladder with six isoforms was used in this study. The anti-T-Tau concentration used was 500,000 pg/mL. The T-Tau protein concentration ranged from 1000 pg/mL to 100,000 pg/mL. DPV measurements showed excellent responses, with a good calibration curve. Thus, a practical tool for simple detection of T-Tau protein, a biomarker of neuro-degenerative disorders, has been successfully developed. This tool could also be extended to detect other biomarkers for neuro-degenerative disorders, such as P-Tau protein and β-amyloid 42.

## 1. Introduction

Neurological degenerative disorders are a frightening health burden. These disorders include Alzheimer’s disease (AD), traumatic brain injury (TBI), different forms of dementia including Parkinson’s disease, and Creutzfeldt–Jacob disease, among others. Both the physical and socio-economic burden of neuro-degenerative disorders are immense. For example, there were currently 5.2 million Americans living with Alzheimer’s disease in 2016, and this number will increase to 23.8 million in 2050. The cost of caring for Alzheimer’s patients in the U.S. is estimated to be $236 billion in 2016 [1]. Furthermore, over 40% of family caretakers report that the emotional stress of their role is high to very high. TBI is a major cause of long-term disability, affecting 100,000 individuals in the U.S. annually. There are no officially established diagnostic methods for TBI. The annual cost of TBI in U.S. is estimated to be $48.3 billion, including $31.7 billion spent on hospital costs, while $16.6 billion goes toward costs associated with fatalities [2].

Researchers have scientifically assessed amyloidopathy and tauopathy and their effects on neuro-degenerative disorders. In amyloidopathy, the formation and characterization of β-amyloid 42 biomolecules are of significance [3,4,5,6]. In tauopathy, the microtubule associate protein, Tau, which binds to and stabilizes microtubules axons, is the protein of focus. The levels of total Tau (T-Tau) and the phosphorylated Tau (P-Tau) are considered useful biomarkers for assessing neuro-degenerative disorders [7,8,9,10,11]. It is generally agreed that any additional tools for the detection of these biomolecules, β-amyloid 42, T-Tau, and P-Tau, will be very useful in evaluating neuro-degenerative disorders. Tau protein is expressed in the neurons of central nervous system and it is critically important in axonal maintenance and axonal transport [12]. T-Tau and P-Tau levels in brain, blood, and cerebrospinal fluid related to neuro-degenerative disorders are well recognized [12,13].

Cerebrospinal fluid (CSF) is commonly chosen as the physiological fluid test medium for the assessment of β-amyloid 42, T-Tau, P-Tau, and others. CSF provides good information for neurological related phenomena [5], but the collection of CSF is an elaborate and complicated process. On the other hand, the collection of a small blood sample (20–30 µL) is a minimally invasive procedure and can be administered relatively simply. Measurements of Tau level in blood as a reliable biomarker for Alzheimer’s disease have been postulated and reported [13,14,15]. The technical challenge will be providing additional tools for the detection of these biomarkers in a simple and accurate manner.

Diagnostic analysis of T-Tau protein in blood samples on brain injury in concussed ice hockey players by the Simoa technique has been reported [16]. This detection was very time consuming, and the blood samples were collected at 1, 12, 36, and 48 h and collected in an ethlenediaminetetracetic acid (EDTA) tube, and an immuno-assay was then employed in the blood sample analysis. The results of this analysis can be very informative, and it is very elaborate, expensive, and time-consuming. Thus, the development of a cost-effective, single-use, disposable in vitro biosensor system which can measure T-Tau in blood accurately can be a first step in providing a practical and useful tool in the assessment of neuro-degenerative disorders, and it is the objective of this study. The bio-recognition mechanism of this biosensor system is based on the antibody and antigen interaction and the effect of a [Fe(CN)_6_]^3−/4−^ redox probe by this interaction. The transduction mechanism is the electrochemical differential pulse voltammetry (DPV) technique. The biosensor was manufactured using a cost-effective roll-to-roll process. Both the working and the counter electrodes were thin gold films, and the reference electrode was a thick-film-printed Ag/AgCl electrode. The Thiol based chemical functionalization step was used to link the antibody to the gold electrode surface. Activation of the carboxylic group on one end of the MPA molecule for the immobilization of anti-T-Tau (antibody of T-Tau) was accomplished by the carbodiimide conjugation technique and showed excellent MPA surface coverage of the biosensor by X-ray photoelectron spectroscopy (XPS) characterization. DPV measurements of T-Tau antigen in both 0.1 M PBS and undiluted human serum showed excellent results. Therefore, the results of this study suggested that a useful tool for the single-use, disposable in vitro measurement of T-Tau to assess neuro-degenerative disorders including Alzheimer’s disease, TBI, and other dementia symptoms in blood serum was feasible.

Diagnosis of T-Tau in one of the neuro-degenerative disorders, Creutzfeldt–Jacob disease, in cerebrospinal fluid used a 1400 pg/mL as the cutoff level of T-Tau in CSF based on Swedish Mortality Registry [17]. Thus, the level of 1400 pg/mL of T-Tau in a physiological fluid is used as the guide for identifying neuro-degenerative disorders.

## 2. Materials and Methods

### 2.1. Apparatus and Reagents

Tau protein, 6 isoforms (Cat. No. T7951), and anti-T-Tau (Cat. No. SAB 5500182) of rabbit monoclonal antibody were both obtained from Sigma Aldrich (St. Louis, MO, USA). Phosphate-buffer saline (PBS) 1.0 M (pH 7.4), human serum, 3-mercaptopropionic acid (MPA), N-(3 dimethylaminopropyl)-N’-ethylcarbodiimide hydrochloride (EDC), and N–hydroxysuccinimide (NHS) were also purchased from Sigma-Aldrich (St. Louis, MO, USA). Potassium hydroxide pellets, concentrated H_2_SO_4_ (95.0 to 98.0 w/w %), and concentrated HNO_3_ (70% w/w %) were obtained from Fisher Scientific (Pittsburgh, PA.). Recombined human beta-amyloid 42 was used in the interference study (Cat. No. ab82795) ABCAM, (Cambridge, MA, USA). All chemicals were used without further purification. A CHI 660C (CH Instrument, Inc., Austin, TX, USA) Electrochemical Workstation was used for DPV and EIS investigations. All experiments were conducted at room temperature. X-ray photoelectron spectroscopy (XPS) was performed by a PHI Versaprobe 5000 Scanning X-Ray Photoelectron Spectrometer.

### 2.2. Design of the Biosensor

**Figure 1:**
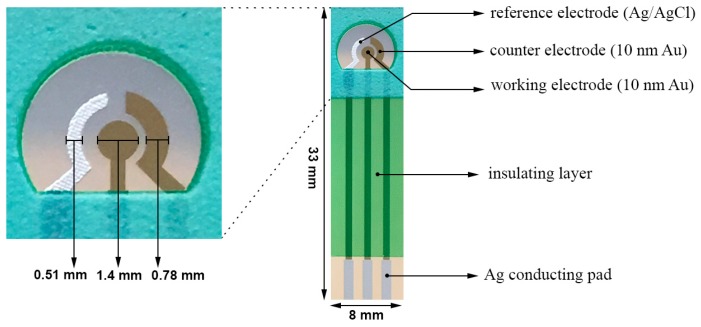
Structure and dimensions of the thin film gold-based T-Tau biosensor prototype [19].

This biosensor prototype for the detection of T-Tau was based on a platform biosensor design. It has a three-electrode configuration. Both the working and counter electrodes were thin gold films 10 nm in thickness. The gold film was deposited by sputtering physical vapor deposition at an atomic level. No binder was employed in any thick-film-printed biosensor including commercially available three-electrode based biosensors. Therefore, the surface of the gold film working electrode was uniform and reproducible. The reference electrode was a thick-film-printed Ag/AgCl electrode. The whole biosensor was formed on a polyethylene terephalate (PET) substrate. Laser ablation technique was used to define the size and dimensions of the biosensor and its electrode elements. The insulator was a thick-film-printed silicon-free dielectric layer made of Nazdar APL 34 ink (Shawnee, KS, USA). This was a cost-effective roll-to-roll fabrication process. In this study, 100 individual biosensors in 4 rows were produced on each PET substrate (355 × 280 mm^2^). Figure 1 shows the structure and actual dimensions of this biosensor. The overall dimensions of an individual biosensor were 33.0 × 8.0 mm^2^. The working electrode area was 1.54 mm^2^ accommodating 20–25 µL of liquid test sample required in this study. A more detailed description of the design and fabrication process of this biosensor has been presented elsewhere [18].

### 2.3. Pretreatment of the Biosensor

Result reproducibility of the biosensor prototype is important for any accurate measurement of the analyte (Tau antigen in this study). Thus, a pretreatment experimental protocol intended to enhance the reproducibility of this thin gold film-based biosensor was established based on previous reports [20,21]. Since the gold film electrodes used in this study were relatively thin, and in order to maintain the integrity of the biosensor prototype, modifications of the pretreatment procedure were needed in comparison to the bulk gold nanoparticles or to the treated particles. This cleaning process would decrease the electrode charge transfer resistance, thus improving the sensitivity of the biosensor. In a typical pretreatment procedure, a row of 5 or 7 biosensors was immersed in a 2 M KOH solution for 15 min. After rinsing with copious amount of DI water, the biosensors were placed in a 20-fold diluted concentrated H_2_SO_4_ solution (95.0 to 98.0 w/w %) for another 15 min. DI water was then used to rinse the biosensor prototypes. The biosensors were then placed in a 20-fold diluted concentrated HNO_3_ solution (70% w/w %) for another 15 min. The biosensors were rinsed once more and then dried by a gentle flow of nitrogen gas. This pretreatment procedure resulted in a significant decrease in electrode charge transfer resistance, enhancing the sensitivity and reproducibility of the biosensor.

### 2.4 Characterization of the Surface Area

**Figure 2:**
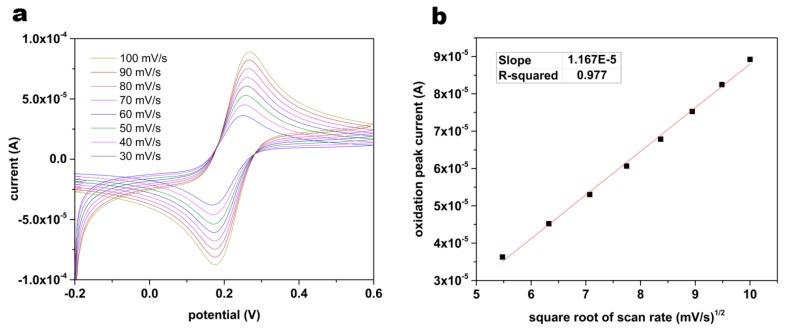
(**a**) Cyclic voltammograms obtained in a solution of K_3_Fe(CN)_6_ and K_4_Fe(CN)_6_, 5 mM in each component, having 0.1 M KCl at scan rates ranging from 30 to 100 mV/s and (**b**) oxidation peak current plotted versus square root of scan rate.

Responses of biosensors were characterized by assessing the stability and reproducibility of the electrochemical surface area. Cyclic voltammograms were obtained at scan rates ranging from 30 to 100 mV/s in a solution of K_3_Fe(CN)_6_ and K_4_Fe(CN)_6_, 5 mM in each component, having 0.1 M KCl (Figure 2a). As presented in Figure 2b, oxidation peak current presented a linear relationship versus the square root of the scan rate. Assuming the diffusion coefficient of ferricyanide ion to be constant, this linear relationship demonstrated the stability of electro-active surface area based on the Randles–Sevcik equation. Using the equation, the calculated electro-active surface area showed less than 2% relative standard deviation from sensor to sensor (n = 3), signifying high reproducibility.

### 2.5. Immobilization of T-Tau Antibody onto the Gold Working Electrode of the Biosensor

In a typical experiment, 5 to 7 biosensors were subjected to surface modification in a single batch. SAM of MPA was employed to covalently immobilize anti-T-Tau on the surface of the gold electrode. The MPA molecule consisted of a thiol functional group at one end, which processed a great affinity to gold, and a carboxylic group at another end which was suitable for bonding covalently to proteins through a peptide bond after an activation procedure. Thiol modification of the gold electrode surface for protein immobilization was a well-developed technique [22,23,24]. The biosensors were immersed in 1 mM solution of MPA in pure ethanol for 24 h, rinsed with DI water, and dried in a steam of N_2_. The MPA-modified biosensors were incubated in 0.1 M PBS (pH = 7.4) containing 0.25 M EDC and 0.05 M NHS for 5 h to activate MPA carboxylic groups. The activated biosensors were then rinsed by 0.1 M PBS and dried by N_2_ flow. 5 µL of 0.05 mg/mL anti-T-Tau was then casted on the sensing area of each biosensor and left to dry overnight at 4 °C. Antibody-immobilized biosensors were rinsed with 0.1 M PBS to remove loosely bonded proteins. The biosensors were then dried again under a steam of N_2_ and stored at 4 °C. This immobilization process was very similar to one described elsewhere [19]. XPS was used to characterize the MPA-SAM coverage of the gold working electrode (results not shown). Identical to our previous study [19], the surface coverage of MPA-SAM was high and the formation of Au–S covalent bond together with the upward orientation of MPA-SAM carboxylic groups, were confirmed. This showed that the immobilization procedure was effective. The XPS results were in agreement with those reported by others [25,26,27,28]. Therefore, the anti-T-Tau bonded biosensor was ready for T-Tau antigen detection.

### 2.6. Differential Pulse Voltammetry (DPV) Measurement

DPV is a well-established electroanalytical technique [29]; however, its applications to biomedical measurement has not been fully exploited. Cyclic voltammetry (CV) and chronoamperometry (CA) are generally used in biomedical measurements. Both CV and CA provide sufficient sensitivity in practical biomedical applications. The required electronic interface for CV and CA are relatively simple. However, DPV applies a series of regular potential pulse superimposed on the potential stair steps. The current is then measured immediately prior to each potential change. Consequently, the charging current can be minimized, resulting in a higher sensitivity. It is based on this technical advantage that DPV was used for the detection of T-Tau using our biosensor system. The anti-T-Tau was first bonded and functionalized as described above. The biosensors were then incubated in solutions of T-Tau with different concentrations for 3 h at room temperature. Antigen solutions were prepared both in 0.1 M PBS and undiluted human serum. After the incubation, biosensors were rinsed with 0.1M PBS removing any unbonded T-Tau. A solution of K_3_Fe(CN)_6_ and K_4_Fe(CN)_6_, 5 mM in each component, was prepared in 0.1 M PBS, 20 µL of this redox probe was dropped to the sensing area of the biosensor, and the DPV measurement then took place.

In this study, an Electrochemical Workstation (CHI 660 model C) was used. The software of the Workstation provided direct experimental setting for the DPV measurement. In a typical measurement, the initial potential was set at −0.3V and the final potential was set at +0.3 V. The potential increase was set at 0.004 V, the amplitude at 0.05 V, and the pulse width at 0.05 s. The pulse period was set at 0.2 s.

## 3. Results and Discussion

### 3.1. Evaluation of the Pretreatment Procedure by Electrochemical Impedance Spectroscopy (EIS)

**Figure 3:**
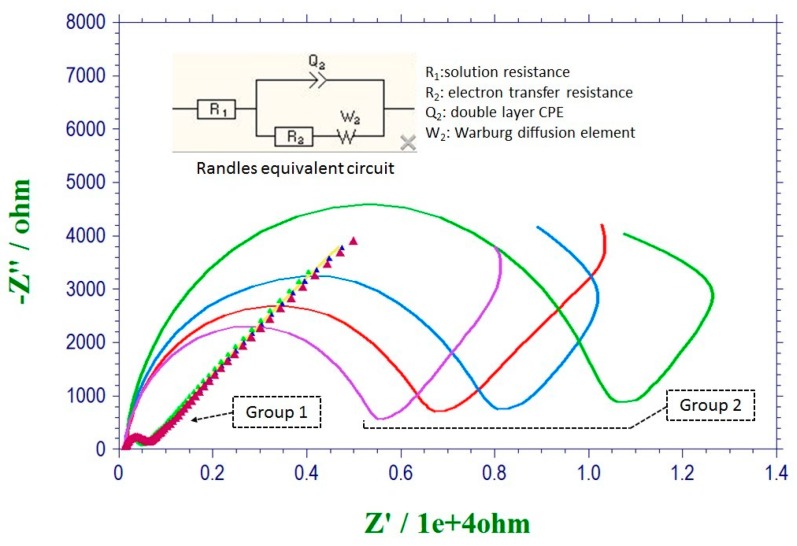
Nyquist plots obtained from EIS in presence of K_3_Fe(CN)_6_/K4Fe(CN)_6_ redox couple for the two groups of sensors (group 1 subjected to cleaning procedure), and equivalent Randles circuit.

In order to validate the enhancement of the sensor response, reproducibility, and electron charge transfer due to the cleaning procedure, electrochemical impedance spectroscopy (EIS) was employed for two groups of sensors consisting of four sensors each. Sensors in Group 1 were subjected to the cleaning protocol described above, whereas sensors in Group 2 were cleaned by ethanol and deionized water (DIW) sequentially. A solution of K_3_Fe(CN)_6_ and K_4_Fe(CN)_6_, 5 mM of each component, was prepared in 0.1 M PBS and used for EIS tests. Twenty microliters of redox couple solution was casted on the sensing area of each sensor for EIS. Figure 3 presents the EIS results obtained for the two groups of sensors in the form of a Nyquist plot using a frequency range of 10^−2^ to 10^4^ Hz with 5 mV voltage amplitude. An equivalent electrical circuit model was fitted to EIS data using EC-lab software. Randles equivalent circuit was selected to model the experimental data. In a typical EIS measurement, the initial potential was set at 0.0 V. The high frequency was set at 10,000 Hz and the low frequency was set at 0.01 Hz. The amplitude was set at 0.005 V and the quiet time was set at 2 s.

Considering the physical structure of the interface, each component in the Randles circuit represents an element in the actual electrode/analyte physical interface. The semicircular region of the Nyquist plots associated with the electron transfer processes was modeled by a parallel circuit representation of a resistor (R_2_) and the constant phase element (CPE). The tail at the lower frequencies indicated the presence of diffusion limited electrochemical processes, represented using the Warburg element (W_2_). The solution resistance was represented by R_1_. Table 1 presents the calculated R_2_ values from Randles model data fitting for all the sensors tested. According to Table 1, the charge transfer resistance (R_2_) that characterizes the interfacial electron transfer resulting from the K_3_Fe(CN)_6_/K_4_Fe(CN)_6_ redox couple, decreases significantly after the cleaning process when comparing the Group 2 sensors to the Group 1 sensors. Moreover, the data scattering, which could be observed for the electrodes in Group 2, was minimized by the cleaning procedure used for the Group 1 sensors. Thus, the EIS test successfully validated the profound effect of this cleaning procedure, demonstrating the excellent reproducibility of the sensors and the decrease in sensor charge transfer resistance.

### 3.2. Measurement of T-Tau Proteins in PBS Solution

The Tau protein ladder, human recombinant (Cat. No. T7951, Sigma Aldrich, St. Louis, MO, USA) was used in this study. Adult brain Tau proteins are varied in size from 352 to 441 amino acids (approximately 36.8 to 45.9 kDa). This protein ladder contained 6 recombinant Tau proteins with molecular masses of 36.8, 39.7, 40.0, 42.6, 42.9, and 45.9 kDa, respectively [30,31]. In the purchased T-Tau protein ladder, 50 µL contained 0.25 µg of each of the six isoforms. In this study, we did not intend to measure each isoform separately and only assessed that the parameter was the total T-Tau protein in the test medium. We used 0.1 M PBS as test medium (pH = 7.4), and dissolved the T-Tau protein ladder in PBS over the range of 1000 pg/mL to 100,000 pg/mL. The anti-T-Tau concentration used was 500,000 pg/mL. This higher antibody concentration was used in order to minimize the possibility of its becoming a rate-limited component in this bio-recognition mechanism. It was feasible to modify and optimize this antibody concentration. This future investigation is beyond the scope of this presentation.

**Figure 4:**
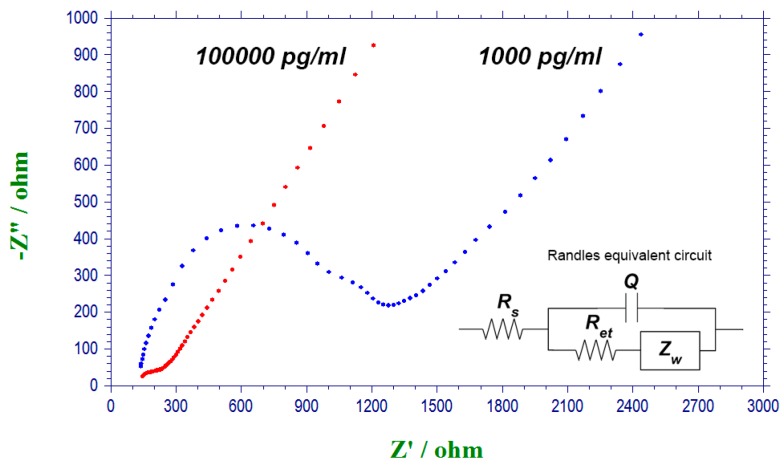
Electrochemical impedance spectroscopy (EIS) Nyquist plots obtained in presence of K_3_Fe(CN)_6_/K_4_Fe(CN)_6_ redox couple for antibody immobilized biosensors incubated in T-Tau solutions for 3 h at room temperature. Antigen solutions were prepared in 0.1 M PBS.

In order to explain the detection mechanism of the T-Tau biosensor, EIS measurements were carried out for biosensors incubated in solutions with different concentrations of T-Tau protein. Figure 4 presents the EIS Nyquist plots obtained in presence of K_3_Fe(CN)_6_/K_4_Fe(CN)_6_ redox couple for antibody immobilized biosensors incubated in T-Tau solutions of 1000 and 100,000 pg/mL. Antigen solutions were prepared in 0.1 M PBS. A Randles equivalent circuit was fitted to the experimental data using EC-lab software. The data obtained from the circuit fitting is presented in Table 2. R_et_ value in Randles equivalent circuit is defined as the resistance to charge transfer of the electrochemical interface.

**Figure 5:**
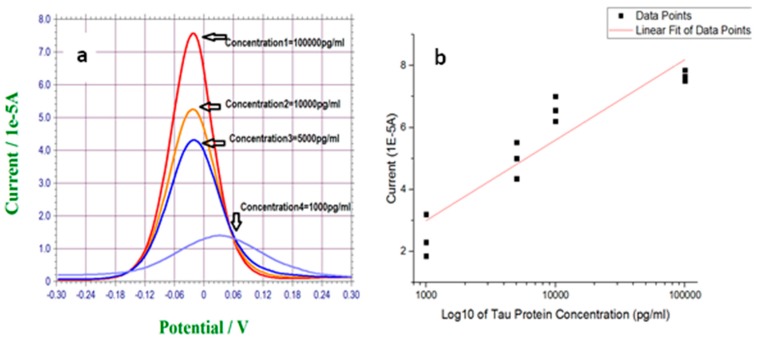
(**a**) Differential pulse voltammetry (DPV) measurement of T-Tau proteins over the concentration range of 1000 pg/mL to 100,000 pg/mL in 0.1 M PBS solution. (**b**) Calibration curve of the DPV outputs and T-Tau protein concentration in 0.1 M PBS solution. Anti-T-Tau concentration is 500,000 pg/mL.

According to Table 2, resistance to charge transfer (R_et_) of the sensing interface decreased significantly from 1154 Ω for the sensor incubated in a T-Tau solution of 1000 pg/mL to 201.2 Ω for the one incubated in a T-Tau solution of 100,000 pg/mL. As reported previously [32], the formation of T-Tau protein film on the surface leads to the development of positive charges, which enhance the charge permeability of the electrode to the negatively charged redox probe of [Fe(CN)_6_]^3−/4−^. Therefore, binding more Tau protein to the surface dramatically decreased the electrode resistance to the charge transfer of [Fe(CN)_6_]^3−/4−^. This phenomenon was exploited as the sensing mechanism in DPV measurements. Figure 5 shows the DPV measurement results of T-Tau proteins in a 0.1 M PBS solution test medium as well as its calibration curve. According to the figure, the anodic peak current associated with one electron transfer reaction of [Fe(CN)_6_]^4-^ to [Fe(CN)_6_]^3-^ was increased by increasing the concentration of T-Tau.

Figure 5a shows the typical DPV measurements of a T-Tau protein concentration of 1000 pg/mL to 100,000 pg/mL. Each measurement was accomplished using a single-use, disposable biosensor. The current outputs obtained were free-of-noise, as demonstrated in Figure 5a. Figure 5b is the calibration curve based on the DPV measurements of multiple experimental runs (n > 3). The axis for the T-Tau protein concentration is in logarithmic scale, covering a wide range of T-Tau concentrations. The calibration curve is a linear least-square-fitting based on experimental data. A linear relationship of Y = 2.6X − 4.7 was obtained, where Y is the current output of the biosensor and X is the T-Tau protein concentration. The R^2^ value of this linear fitting is 0.85. One must recognize that this detection covered a very large concentration range of T-Tau protein and the modification step of the biosensor was carried out individually and manually. Thus, the uniformity of each biosensor was not 100% identical. Refinement could further enhance this linear relationship between the current outputs of the biosensor and the T-Tau concentration.

### 3.3. Measurement of T-Tau Proteins in Undiluted Human Serum

DPV measurements for different T-Tau protein concentrations in undiluted human serum were also carried out in this study. Human serum (Cat No.3667, Sigma Aldrich, St. Louis, MO, USA) was used to prepare antigen solutions.

**Figure 6:**
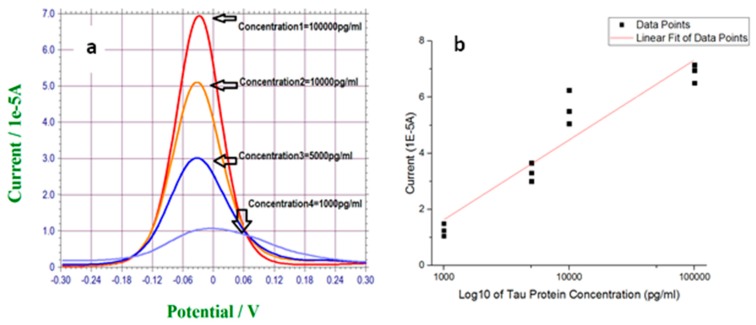
(**a**) DPV measurement of T-Tau proteins over the concentration range of 1000 pg/mL to 100,000 pg/mL in undiluted human serum. (**b**) Calibration curve obtained from DPV measurements for T-Tau protein concentration range of 1000 to 100,000 pg/mL in undiluted human serum. Anti-T-Tau concentration is 500,000 pg/mL.

Figure 6a shows the typical DPV measurements of various T-Tau protein concentrations in the serum. The T-Tau protein concentration was tested between 1000 pg/mL and 100,000 pg/mL. Similar to the test in 0.1 M PBS, a single-use, disposable biosensor was used for each measurement. Figure 6b shows the corresponding calibration curve of DPV measurements in serum based on results shown in Figure 6a with n = 3. The anti-T-Tau concentration used in this phase of study was 500,000 pg/mL. Figure 6b yields a least-square fitted calibration equation of Y = 2.8X − 6.9, where Y is the current outputs of the biosensor and X is the T-Tau concentration in blood serum. The R^2^ value of this equation was 0.88. It is feasible to optimize operational parameters further in this T-Tau detection system, such as the antibody concentration, the range of the detecting T-Tau proteins, concentration, and others. Our purpose of this presentation is to demonstrate that the basic designed biosensor with DPV transduction mechanism can be effectively used for T-Tau protein detection.

### 3.4. Interference Study of T-Tau Proteins Measurement of This Biosensor

**Figure 7:**
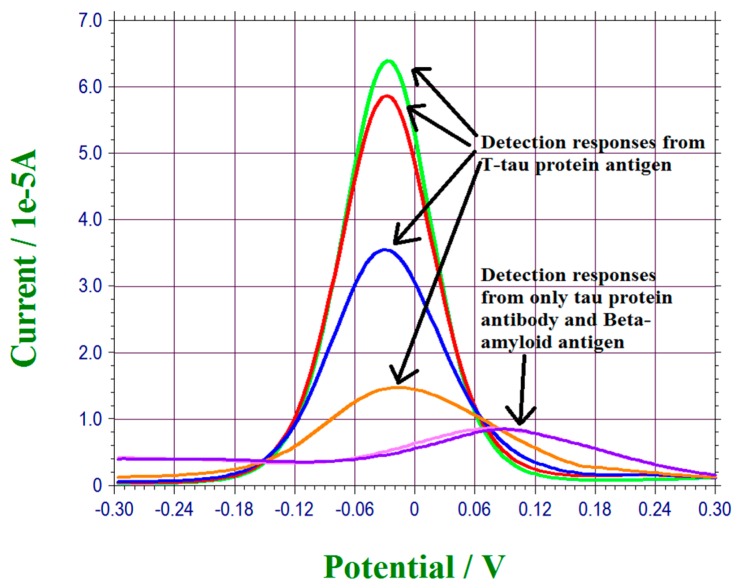
Interference study using β-amyloid 42 of 50,000 pg/mL as the biomarker.

The bio-recognition mechanism of this biosensor was based on the interaction of the antibody and antigen of T-Tau protein, and this detection mechanism was very specific. However, in order to examine any potential interference, β-amyloid 42, another important biomarker of neuro-degenerative disorders, was used in this interference study. The T-Tau detection biosensor was prepared as described previously as in Section 2.4. The anti-T-Tau concentration used was maintained at 500,000 pg/mL Recombined human β-amyloid 42 at a concentration of 50,000 pg/mL with an incubation time of 2 h, identical to a previous study in PBS, was then used in this study. Figure 7 shows the testing results. Both the β-amyloid 42 antigen and the zero concentration of T-Tau PBS solution show the same base line as comparing to other T-Tau protein-contained PBS solutions as presented in Figure 5. This study confirmed positively that this T-Tau biosensor was specific to T-Tau protein only.

## 5. Conclusions

A cost-effective single-use, in vitro biosensor for the detection of a biomarker of neuro-degenerative disorder, T-Tau protein, has been designed, manufactured, and evaluated in phosphate-buffer saline and undiluted human serum. DPV was used as the measurement technique. Measurements of T-Tau protein in both 0.1 M PBS and undiluted human serum over the concentration range of 1000 pg/mL to 100,000 pg/mL showed excellent results and good linearity of the calibration curves. This biosensor platform technology can be further optimized and can be applied to detect other biomarkers of neuro-degenerative disorders, including P-TV.au protein and β-amyloid 42.

## Figures and Tables

**Table 1:** Calculated R2 (charge transfer resistance) values from Randles model data fitting.

	Sensor #1	Sensor #2	Sensor #3	Sensor #4
Group 1	198 Ω	200 Ω	201 Ω	204 Ω
Group 2	6150 Ω	6913 Ω	7941 Ω	11346 Ω

**Table 2:** Data obtained from Randles equivalent circuit modeling of EIS Nyquist plots in Figure 4.

T-Tau (pg/mL)	*Q*(*μF*)	$Z_{w}\left(\Omega \right)$	*R_et_*(*Ω*)	*R_s_*(*Ω*)
1000	1.33	1480	1154	125
100,000	120.8	1865	201.2	68.8
